# The Role of Microbial Mats in the Removal of Hexavalent Chromium and Associated Shifts in Their Bacterial Community Composition

**DOI:** 10.3389/fmicb.2020.00012

**Published:** 2020-01-29

**Authors:** Raeid M. M. Abed, Mary Shanti, Thirumahal Muthukrishnan, Zayana Al-Riyami, Bernhard Pracejus, Daniel Moraetis

**Affiliations:** ^1^Department of Biology, College of Science, Sultan Qaboos University, Muscat, Oman; ^2^Earth Science Department, College of Science, Sultan Qaboos University, Muscat, Oman; ^3^Department of Applied Physics and Astronomy, College of Sciences, University of Sharjah, Sharjah, United Arab Emirates

**Keywords:** microbial mats, hexavalent chromium, MiSeq, quarry sumps, bacterial communities

## Abstract

Microbial mats are rarely reported for chromium-polluted ecosystems, hence information on their bacterial diversity and role in chromium removal are very scarce. We investigated the role of nine microbial mats, collected from three quarry sumps of chromium mining sites, in the removal of hexavalent chromium [Cr(VI)]. Bacterial diversity in these mats and community shifts after incubation with Cr(VI) have been investigated using MiSeq sequencing. In nature, a chromium content of 1,911 ± 100 mg kg^–1^ was measured in the microbial mats, constituting the third highest source of environmentally available chromium. The mats were able to remove 1 mg l^–1^ of Cr(VI) in 7 days under aerobic conditions. MiSeq sequencing of the original mats yielded 46–99% of the sequences affiliated to Proteobacteria, Firmicutes and Actinobacteria. When the mats were incubated with Cr(VI), the bacterial community shifted in the favor of Alphaproteobacteria and Verrucomicrobiae. We conclude that microbial mats in the quarry sumps harbor diverse microorganisms with the ability to remove toxic Cr(VI), hence these mats can be potentially used to remove chromium from polluted waters.

## Introduction

The use of heavy metals such as chromium has remarkably increased over the last decades because of rapid industrialization and urbanization. Chromium is used in metal processing, leather tanning, cement dyeing, wood preservation, paints, textile and canning industries (Wang and Xiao, 1995; Pattanapipitpaisal et al., 2001a, b; Narayani and Shetty, 2013). High volumes of chromium-contaminated effluent wastewaters are produced from these industries and the discharge of chromium into aquatic ecosystems has become a serious environmental threat. Among other heavy metals, chromium is considered to be highly toxic and the consumption of drinking water with high chromium content can cause death (Shukla et al., 2012). Humans exposed to chromium-polluted groundwater or seawater were affected by skin, gastrointestinal, respiratory, reproductive and genetic disorders leading to cancer (Shukla et al., 2012; Narayani and Shetty, 2013). Accumulation of chromium in plants and animals induced DNA damage and intense oxidative stress within cells resulting in genotoxic and mutagenic effects to these organisms (Narayani and Shetty, 2013). In the environment, chromium can be found in a hexavalent form[Cr(VI)], which is highly soluble and readily available, or in a trivalent form [Cr(III)], which is less soluble and much less toxic (Francisco et al., 2002). Because of the diverse applications of chromium and its drastic impacts on humans and environment, there is an urgent need to design strategies to remove Cr(VI) from effluents. Recently, a number of bacterial strains have been isolated with the ability to reduce the highly toxic Cr(VI) to the less toxic Cr(III) (Mishra et al., 2012; Long et al., 2013; Voica et al., 2016).

Ophiolite rocks, such as harzburgites, dunites, and even gabbros/basalts, are considered as an important natural source of Cr(VI) (Oze et al., 2004). Such rocks are widespread in the Sultanate of Oman, with one of the most complete and extensive oceanic crust exposure (Rabu et al., 1986). Rocks in oceanic crust have much higher chromium content (ca. 2000 mg kg^–1^) compared with the continental crust (ca. 20 mg kg^–1^) (Daly et al., 1966). The weathering of silicate minerals in ophiolite rocks releases Cr(III), which is then oxidized to detectable amounts of Cr(VI) in groundwater (Oze et al., 2004; Morrison et al., 2009; Moraetis et al., 2012; Kelepertzis and Stathopoulou, 2013). The chromium geochemical cycle and especially the Cr(III) conversion into the more mobile Cr(VI) has been mainly related to Mn oxides (Stanin, 2005). However, the role of the biology in this geochemical cycle has been mainly described as a convertor of Cr(VI) to Cr(III) (Palmer and Puls, 1994).

In the past decades, a rise in the need for chromite export has increased the number of chromite mining operations in several locations around Oman. The mining practices include solely open pit excavations (quarry sumps) and the bulk rocks rich in chromite are exported without prior enrichment or other beneficiation processes. In these quarry sumps, different types of microbial mats, either attached to the sediment or floating at the water surface, have been observed. Such mats have been rarely reported for chromium-contaminated sites and so far, few studies have been performed to investigate their microbial community composition and their possible role in chromium removal (Bender and Phillips, 2004; Shukla et al., 2012). In previous studies, microbial mats have been shown to sequester and/or precipitate metals, including Cr(VI) by surface adsorption or by changing the surrounding chemical environment (Bender and Phillips, 2004; Shukla et al., 2012). Similar findings were also reported pertaining to indigenous soil microorganisms (Kamaludeen et al., 2003). However, continuous accumulation of chromium was shown to reduce microbial growth and induce a shift in the microbial community structure, not only in soil but also in freshwater biofilms (Mona et al., 2011; Focardi et al., 2013; Golby et al., 2014).

This study was undertaken to investigate the ability of chromium-contaminated microbial mats from excavation quarry sumps to remove Cr(VI) and to reveal the composition of their bacterial communities. This basic research will promote our understanding of what kind of microorganisms survive in chromium-impacted microbial mats and what mechanisms they employ to remove Cr(VI) from the environment. Such knowledge can eventually help in designing strategies to decontaminate Cr(VI) polluted environments.

## Materials and Methods

### Sampling Sites

**FIGURE 1:**
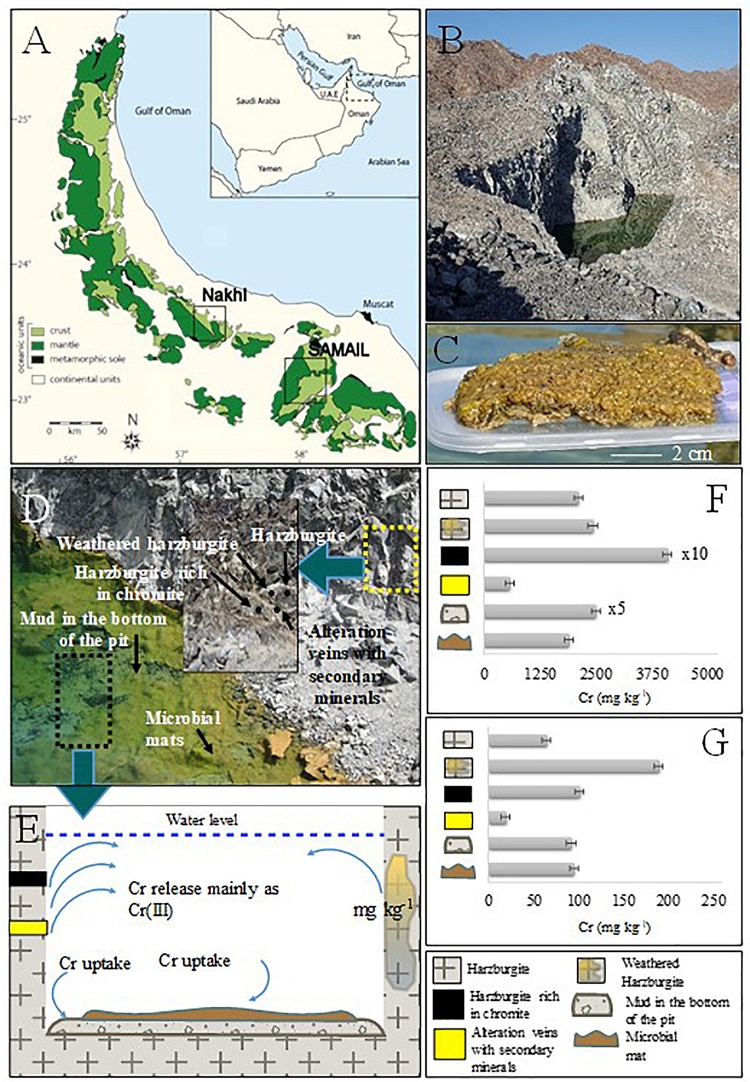
**(A)** Geological map showing mantle and crust rocks in Oman, investigated localities are also shown. **(B)** Nakhl quarry sump. **(C)** Collected microbial mat. **(D)** Close view of the quarry sump with the areas of solid and microbial mat sampling. The thick cover of microbial mat is shown. **(E)** Schematic graph representing Cr cycle in the quarry sump. **(F)** Bulk Cr(total) content in the collected solid samples from Nakhl quarry sump. **(G)** Environmental available Cr(total) with EPA3050.

Microbial mats and water samples were collected from quarry sumps in three abandoned chromite mining sites (termed as Nakhl-1, Nakhl-2, and Samail; Figures 1A,B) in February 2017. The sites Nakhl-1 and Nakhl-2 are 500 m apart and are located near the village of Nakhl, 70 km north-east of the capital city of Muscat, while the Samail site is 50 km south-east of Muscat (Figure 1A). Three different mats (i.e., A, B, and C) were collected from each site, some were floating while others were benthic. At Nakhl-1 site, the mats A and B were similar in appearance and were found floating at the water surface. Mat C was submerged and attached to the sediment. A vibrant green color at the bottom of all three mats could be seen, which phase into a lighter brown at the top (Figure 1C). The texture was spongy and entwined and the mats remained intact when sampled. The mats collected from Nakhl-2 site were all submerged and loosely attached to rocks. These mats were gelatinous and fragile, easily broken up into smaller pieces when collected. The mats were brown and contained many small rock fragments. Unlike at Nakhl-2 site, all the mats collected from Samail were floating at the water surface and had a consolidated structure. These mats had a slimy gelatinous surface texture and remained intact when sampled. Mat A and B had a deep green color and appeared healthy whereas mat C was brownish in color and showed signs of decay. Based on light microscopy, a total of 17 taxa of phototrophic microorganisms (eukaryotic algae and diatoms) were detected in all mats (Supplementary Figure S1). All samples were collected in sterile plastic containers and transferred immediately to the laboratories of Sultan Qaboos University (SQU). Samples for molecular work were stored in a freezer at −20°C until analysis while live samples were directly used for the Cr(VI) removal experiments (see below).

Water samples were collected from the three chromite mines. Physical and chemical parameters such as pH, temperature, electrical conductivity (EC), oxidation and removal potential (ORP) and dissolved oxygen (DO) were measured with Aquaprobe AP-800 from AquaRead. The water samples were filtered with Whatman paper of 0.45 μm pore size. A fraction of the water sample (50 ml) was acidified with 3% HNO_3_ for further analysis with ICP-MS (Bruker, Aurora M90) while another fraction (450 ml) was preserved in a cooler for nutrient analysis with a portable LaMotte photometer.

### Chromite Distribution in Nakhl-1 Quarry Sump

In order to cover all the possible sources of leachable (i.e., environmentally available) chromium, samples were collected only from Nakhl-1 quarry sump. The mineralogical analysis was restricted to Nakhl-1 quarry sump because the three sumps are considered of the same rock unit with the same lithology of harzburgite with dunite in concordant lenses (Rabu et al., 1986). Harzburgite, weathered harzburgite, alteration veins of secondary minerals, harzburgite rich in chromite, and mud deposits from the bottom of the sump were sampled. All solid samples were dried and ground to a size of less than 100 μm. X-ray diffraction (XRD) was performed for mineralogical analysis with the X-Pert Pro instrument (Panalytical). X-ray fluorescence (XRF) was used for bulk chemical analysis of major, minor, and trace elements with Niton XL3t Goldd (Thermo Fischer). Finally, leaching of environmentally available metals was performed for all solid samples following EPA3050B (U.S.EPA, 1996).

### Cr(VI) Removal Experiments

Different incubations were carried out to reveal the role of microbial mats in the removal of Cr(VI) and to identify the optimum conditions at which maximum removal occurred. These experiments were performed in 160 ml glass bottles. Each bottle received 30 ml of filter sterile site water, 0.5 g of the mat and 1 mg l^–1^ of Cr(VI). Pre-prepared packets provided by TRACE–HT22 chromium hexavalent batch code R09A, were used to prepare the Cr(VI) stock solution. The removal of Cr(VI) was assessed with a colorimetric analysis at 540 nm of the complex developed by binding of Cr(VI) with diphenylcarbizide under acidic conditions according to EPA 7196A (U.S.EPA, 1992). The mats from the three sites were incubated in triplicates in the dark under aerobic conditions to reveal the role of aerobic bacteria in Cr(VI) removal. Each bottle was wrapped with aluminum foil to prevent the photosynthetic activity by phototrophs. Based on the results of this experiment, five mats (i.e., Nakhl-1A and C, Nakhl-2A and C, and Samail-A) that showed the highest rates of Cr(VI) removal were chosen for further experiments. Similar setups as in the dark experiment were maintained, but were incubated aerobically under 12 h light and 12 h dark to reveal the role of phototrophic bacteria in Cr(VI) removal. The bottles incubated in the light were illuminated using cool white LED lamps (100W) at an intensity of 1550 Lux as measured using Light Meter (Extech, United States). Control bottles containing only mats but without Cr(VI), or Cr(VI) but without mats were maintained. Additional controls were maintained for Nakhl-1 and Nakhl-2 samples after autoclaving mats at 121°C for 20 min prior to incubation to account for the passive sorption of Cr(VI) in aerobic conditions. All these incubations were performed in triplicate and at 30°C. Water samples were collected at different time intervals (i.e., 2, 4, 6, 8, and 10 days) and Cr(VI) concentrations were monitored using the aforementioned method. To confirm the Cr(VI) removal in the above experiments, the water samples collected at the end of the experiment in the two sites which showed the highest Cr(VI) removal were analyzed for their total chromium content using inductively coupled plasma-optical emission spectrometry (ICP-OES). The amount of Cr(III) present in the solution was calculated by subtracting the colorimetrically estimated amount of Cr(VI) from the total amount of chromium measured using ICP-OES. Furthermore, the presence of Cr in the algal mats and not by any type of mineral precipitation was analyzed using scanning electron microscopy (SEM) and energy dispersive x-ray spectroscopy (EDX; Jeol JSM-7600). Approximately 0.05–0.1 g of the Nakhl-1 and Nakhl-2 mat samples from the dark incubations were air-dried on aluminum stubs, vacuum-coated with platinum and analyzed using SEM (JEOL-JSM 7600F, United States) operating at 25 kV with a 7.4 mm working distance and a total magnification range of 2000× to 45,000×.

### Bacterial Community Analysis

Bacterial diversity in the original mats samples was investigated using high throughput Illumina MiSeq sequencing of 16S rRNA genes. Mat samples at the end of the dark incubation experiments were also analyzed using Illumina MiSeq to monitor shifts in the microbial community composition. DNA was extracted from the mats using skim milk protocol (Volossiouk et al., 1995). Purified DNA extracts were then submitted to Molecular Research MR DNA laboratory (Shallowater, TX, United States)^1^ for illumina MiSeq sequencing of the bacterial 16S rRNA genes using the primers 341F (5′-CCTACGGGNGGCWGCAG-3′) and 805R (5′-GACTACHVGGGTATCTAATCC-3′) with barcode on the forward primer (Klindworth et al., 2013). A HotStarTaq Plus Master Mix Kit (Qiagen, United States) was used with the following PCR conditions: 94° for 3 min, followed by 28 cycles of 94° for 30 s, 53° for 40 s and 72° for 1 min, after which a final elongation step at 72° for 3 min was performed. After amplification, PCR products were checked in 2% agarose gel to determine the success of amplification and the relative intensity of bands. Multiple samples were pooled together in equal proportions based on their molecular weight and DNA concentrations. Pooled samples were purified using calibrated AMPure XP beads. Then, the pooled and purified PCR products were used to prepare a DNA library by following Illumina DNA library. Sequencing was performed on a MiSeq following the manufacturer’s guidelines. Sequence data were processed using MR DNA analysis pipeline (MR DNA, Shallowater, TX, United States). In summary, sequences were joined, depleted of barcodes then sequences <150 bp and sequences with ambiguous base calls were removed. Sequences were denoised, operational taxonomic units (OTUs) were generated and chimeras were removed. OTUs were defined by clustering at 3% divergence (97% similarity). Final OTUs were taxonomically classified using BLASTn against a curated database derived from RDPII and NCBI^2^ ^,3^.

Alpha diversity indices (OTU richness, Chao-1 and ACE) were calculated using the Mothur software (Schloss et al., 2009). Cluster analysis based on the sequences data was performed using PAST program to examine for significant differences in microbial communities in the original and treated mats. OTU partitioning was used to find out the percentage of OTUs that are specific for each site or for each treatment (i.e., with and without chromium).

## Results

### Physicochemical Characteristics of the Water in the Sampling Sites

The water temperature at the sampled quarry sumps was between 21.0 ± 0.1 to 25.1 ± 0.1°C while pH was alkaline, in the range of 9.05 to 9.44 (Table 1). The electrical conductivity (EC) and nutrients such as nitrate were higher in the Nakhl-1 than in Nakhl-2 and Samail quarry sumps. Dissolved oxygen (DO) and oxygen removal potential (ORP) showed also higher values in Nakhl-1 than in Nakhl-2 and Samail quarry sumps. Total chromium in both Nakhl-1 and Nakhl-2 sites was twofold higher than in the Samail site and most of this chromium was in the form of Cr(VI) (Table 1).

**TABLE 1:** Physical and chemical characteristics of water samples collected from Nakhl and Samail quarry sumps.

Parameters	Nakhl-1 quarry	Nakhl-2 quarry	Samail quarry
	sump	sump	sump
Latitude	N 23°23.9788′	N 23°24.0079′	N 23°10.7567′
Longitude	E 57°44.5489′	E 57°44.5321′	E 57°56.9463′
pH	9.05	9.44	9.16
Temperature (C°)	25.1 ± 0.1	21.0 ± 0.1	23.4 ± 0.1
EC (μS/cm)	1793 ± 20	862 ± 20	623 ± 20
ORP (mV)	210 ± 25	−34 ± 25	173 ± 25
DO (mg l^–1^)	5.4 ± 0.2	2.6 ± 0.2	2.9 ± 0.2
Chloride (mg l^–1^)	82.9 ± 0.1	78 ± 0.1	44 ± 0.1
Ammonium (mg l^–1^)	1.0 ± 0.05	0.52 ± 0.05	0.60 ± 0.05
Nitrate (mg l^–1^)	27.0 ± 0.1	0.50 ± 0.1	0.30 ± 0.1
Phosphate (mg l^–1^)	ND	0.63 ± 0.01	0.12 ± 0.01
Sulphate (mg l^–1^)	57.9 ± 0.01	17.44 ± 0.01	ND
Total hardness (mg l^–1^)	1651 ± 5	275 ± 5	288 ± 5
Ca (mg l^–1^)	14.0 ± 0.01	2.45 ± 0.01	4.60 ± 0.01
Mg (mg l^–1^)	ND	0.13 ± 0.01	31.7 ± 0.01
Fe (mg l^–1^)	0.17 ± 0.01	0.10 ± 0.01	0.21 ± 0.01
Mn (mg l^–1^)	ND	0.13 ± 0.01	ND
Ni (mg l^–1^)	0.02 ± 0.01	ND	ND
Cr total (μg l^–1^)	46.1 ± 0.05	50.1 ± 0.05	20.3 ± 0.05
Cr(VI) (μg l^–1^)	40 ± 1	44 ± 1	20.3 ± 1

*EC: Electrical Conductivity; ORP: Oxygen reduction potential; DO: Dissolved oxygen; ND: Not detected.*

### Chromium in the Rocks and Mats of Nakhl-1 Quarry Sump

The mineralogical analysis of rocks/muds in Nakhl-1 quarry sump showed a variety of minerals (Supplementary Table S1) that can host chromium in their structure. The analysis also showed that there are different sources contributing chromium in the groundwater (Figure 1D). Harzburgite contained olivine, serpentine, and pyroxene, while the secondary minerals in veins were mainly serpentine, magnesite, and dolomite (Supplementary Table S1). The harzburgite rich in chromite contained olivine and clinochlore and the mud from the bottom of the quarry sump showed a variety of minerals like dolomite, serpentine, olivine and magnetite (Supplementary Table S1). The highest total chromium content in the solids was detected within harzburgite rich in chromite (41,124 ± 100 mg kg^–1^), as expected, followed by the mud sample from the bottom of the quarry sump (12,547 ± 100 mg kg^–1^) (Figure 1F and Supplementary Table S1). The bulk content of chromium in the microbial mats (1,911 ± 100 mg kg^–1^) was close to that of the harzburgite (2,120 ± 100 mg kg^–1^) and the weathered harzburgite (2,445 ± 100 mg kg^–1^; Figure 1F and Supplementary Table S1). The highest amount of environmentally available chromium was measured in the weathered harzburgite and the mud from the bottom of the sump (Figure 1G and Supplementary Table S1). Microbial mats were the third highest source of environmental available chromium (Supplementary Table S1).

### Cr(VI) Removal

**FIGURE 2:**
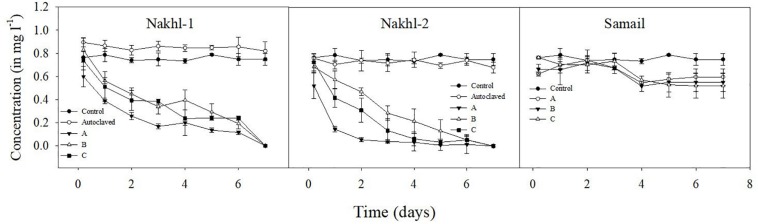
Cr(VI) removal by nine microbial mats collected from Nakhl-1, Nakhl-2, and Samail quarry sumps after incubation in the dark under aerobic conditions. The absence of passive adsorption of Cr(VI) into mat represented by data shown for one autoclaved mat each from Nakhl-1 and Nakhl-2. Each mat was incubated in triplicates. Error bars represent ±standard deviation (*n* = 3).

**FIGURE 3:**
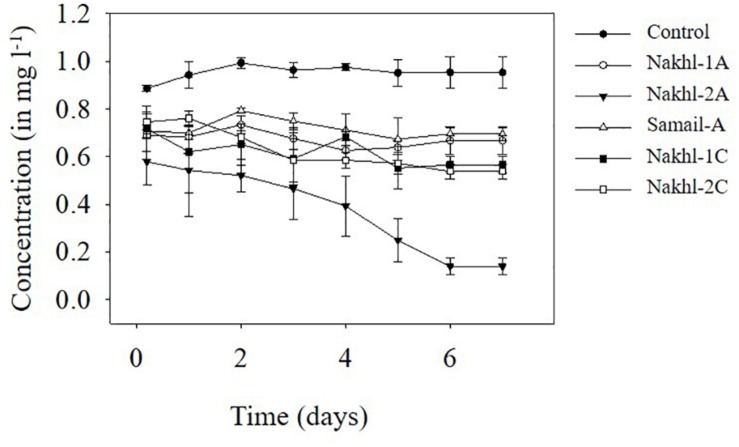
Cr(VI) removal by five selected microbial mats from Nakhl-1 (mats A and C), Nakhl-2 (mats A and C) and Samail (mat A) quarry sumps incubated aerobically in 12 h light/12 h dark. Each mat was incubated in triplicates. Error bars represent ±standard deviation (*n* = 3).

Spectroscopic analysis of Cr(VI) after 7 days of incubation in the dark under aerobic conditions revealed a complete removal by mat samples from Nakhl-1 but lesser in the mats from Nakhl-2 and Samail (Figure 2). In the control treatment without any mat material or with autoclaved mat, Cr(VI) concentration did not vary throughout the 7 days of incubation (Figure 2). The removal rate of Cr(VI) by Nakhl-1 and Nakhl-2 mats reached 0.09 ± 0.03 mg l^–1^ d^–1^. Incubation of the five selected mats in 1 mg l^–1^ Cr(VI) under 12h light and 12 h dark showed the inability of these mats to decrease Cr(VI), except in the case of Nakhl-2A mat (Figure 3). Cr(VI) concentration in this mat decreased from 0.6 to 0.14 mg l^–1^ after 7 days of incubation.

**FIGURE 4:**
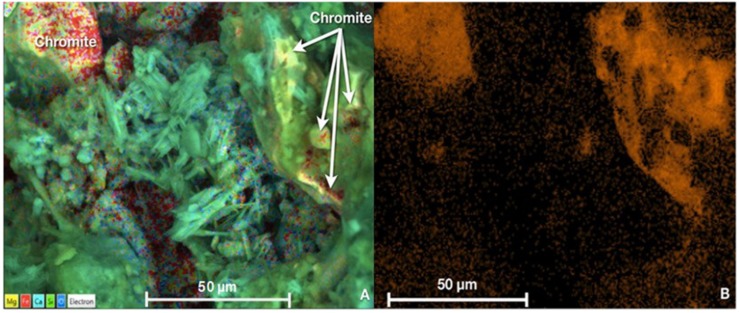
**(A)** SEM micrograph showing the presence of chromite grains or fragments (top left corner) and low levels of Cr homogenously diffused in the EPS of the mats (indicated by white arrows in top right corner) **(B)** Variations in the orange hue intensity indicative of diffused Cr concentrations in the mat as obtained by EDX analysis. Scale bar representative of length of 50 μm in all cases.

The ICP-OES analysis showed that the remaining total Cr in Nakhl-1 sitewater was from 0.02 to 0.04 mg l^–1^ which corresponded to 96–98% of Cr(VI) removal (Supplementary Table S2). Approximately 2–4% of the remaining Cr left in the solution was present as Cr(III) (Supplementary Table S2). Similarly, a removal of 83–88% of Cr(VI) was detected in the Nakhl-2 sitewater where Cr(III) was not detected rather Cr(VI) (Supplementary Table S2). The SEM and EDX analyses demonstrated two types of chromium occurrence in the mats (Figures 4A,B). Certain areas on the mat showed the presence of well-defined fragments of chromite (FeCr_2_O_4_), which was not associated with other minerals and originated from the quarry sump by being bound to the mat prior to sampling (Figure 4A). A more diffuse distribution of Cr was detected within the extracellular polymeric substances (EPS) of the mat (Figures 4A,B). EDX analyses of these Cr-diffused areas without any correspondence to the incorporated chromite fragments showed low levels of chromium ranging from 0.3–0.7 mg l^–1^.

### Bacterial Diversity in the Original Mats

**FIGURE 5:**
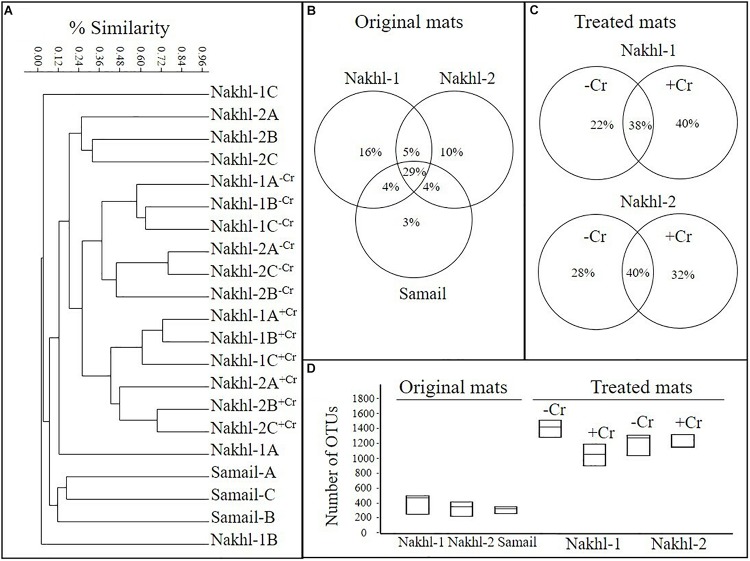
**(A)** Cluster analysis, based on MiSeq sequencing data, showing similarities among the bacterial communities of the original microbial mats collected from the three studied quarry sumps and their communities after treatment with and without 1 mg l^–1^ Cr(VI). **(B,C)** Partitioning of OTUs based on different sites and based on with and without Cr(VI). **(D)** Average number of OTUs in the original and treated mats.

OTU richness in the original mats was comparable, with the highest value in Nakhl-1A mat (507 OTUs) and the lowest in Nakhl-2B mat (i.e., 237 OTUs; Table 2). Cluster analysis based on Bray-Curtis dissimilarities showed that bacterial communities from the triplicate mats of the same sampling site clustered together, except in Nakhl-1 samples (Figure 5A). When all detected OTUs were compared among the three sites, 29% were common OTUs (Figure 5B), whereas 16, 10, and 3% were ubiquitous OTUs of the mats from Nakhl-1, Nakhl-2 and Samail, respectively (Figure 5B).

**TABLE 2:** MiSeq sequencing and bacterial diversity estimators for the original microbial mats from chromium mining quarry sumps and after incubating them with and without 1 mg l^–1^ Cr(VI).

Quarry	Mat	Total No. of	No. of	Chao-1	ACE	SSO	DSO
sump		sequences	OTUs^∗^				
**Original mats**							
Nakhl-1	1A	192036	507	666	686	8.8	12.3
	1B	113094	479	625	634	4.1	8.0
	1C	45479	265	400	431	0.7	1.5
Nakhl-2	2A	207367	425	593	607	4.6	5.1
	2B	94925	237	377	390	2.2	4.4
	2C	96054	362	491	514	1.9	3.8
Samail	3A	112388	272	405	403	0.5	1.3
	3B	136950	364	495	506	0.9	1.8
	3C	72039	332	437	446	0.6	1.5
**Treated mats**							
Nakhl-1	1A (−)	44549	1295	2607	2730	13.5	8.3
	1B	80665	1519	2638	2671	11.0	10.3
	1C	67514	1433	2524	2551	10.7	10.4
	1A (+)	44629	1061	2230	2306	10.3	7.5
	1B	66699	905	1916	2051	9.9	7.6
	1C	44209	1205	2215	2240	10.0	7.9
Nakhl-2	2A (−)	100705	1050	2075	2123	9.6	7.8
	2B	102991	1323	2507	2578	12.2	10.0
	2C	84721	1286	2479	2529	11.3	9.6
	2A (+)	88034	1330	2385	2523	12.8	11.9
	2B	69559	1154	2281	2423	9.7	7.4
	2C	40452	1164	2164	2168	9.4	8.4

*^∗^Operational taxonomic unit at 3% sequence dissimilarity based on equal subsets of sequences for all samples, Chao-1 is based on rare OTUs in a given sample and ACE is abundance-based coverage. SSO refers to singletons sequences that were observed once and DSO refers to doubletons sequences that were observed twice.*

**FIGURE 6:**
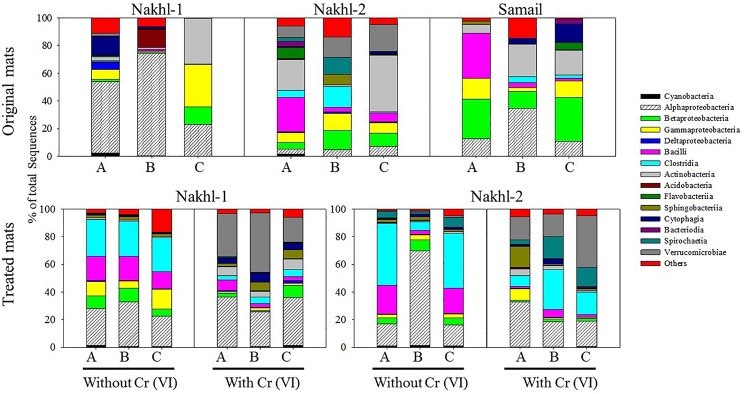
The relative abundance (%) of major Phyla and classes of bacteria present in the original and treated mats from Nakhl-1, Nakhl-2, and Samail quarry sumps. A, B and C represent the three different mats collected from each site.

**FIGURE 7:**
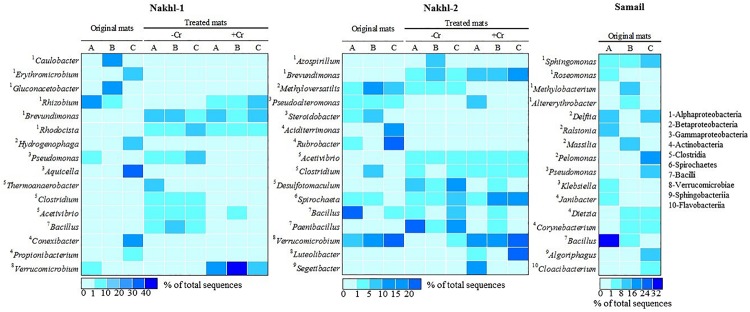
A heatmap showing the relative abundance (%) of the most dominant bacterial genera detected in the original mats and their shift after incubation with and without Cr(VI).

The original mats exhibited bacterial community differences within each site and between the different sites (Figure 6). At Nakhl-1, mat A was dominated by Alphaproteobacteria and Cytophagia (65% of the total number of sequences), while mat B was dominated by Alphaproteobacteria and Acidobacteria (87%) (Figure 6). Alphaproteobacterial sequences in these mats mainly belonged to the genera *Rhizobium* and *Gluconacetobacter* (Figure 7). Nakhl-1C mat was mainly dominated by Gammaproteobacteria and Bacilli (63%) constituting the genera *Aquicella* and *Bacillus*, respectively (Figure 7). Unlike in Nakhl-1 mats, Actinobacteria, Bacilli and Verrucomicrobiae were prevalent (20–70%) in all Nakhl-2 mats (Figure 6), with most sequences belonging to *Rubrobacter*, *Aciditerrimonas*, *Bacillus* and *Verrucomicrobium*, respectively (Figure 7). In Nakhl-2B mat, Clostridia, Gammaproteobacteria and Betaproteobacteria constituted 45% of all sequences (Figure 6). Most of their sequences fell into the genera *Clostridium, Pseudoalteromonas* and *Methyloversatilis*, respectively (Figure 7). At Samail, the most detectable groups were Bacilli (32%) in mat A including sequences mostly belonging to the genus *Bacillus* and Actinobacteria (18–23%) in mats B and C (Figure 6). Alpha-, Beta-, and Gammaproteobacteria accounted for 45–50% of total number of sequences in all Samail mats (Figure 6).

### Post-incubation Bacterial Community Shifts

Cluster analysis clearly demonstrated a shift in the bacterial community composition after incubating the mats in the dark with and without Cr(VI) (Figure 5A). All the triplicate mats from either Nakhl-1 or Nakhl-2 formed separate clusters when incubated without Cr(VI) and were distinct from those that included Cr(VI)-treated mats (Figure 5A). The shared OTUs between the mats with and without Cr(VI) constituted 38% and 40% of total OTUs in Nakhl-1 and Nakhl-2, respectively (Figure 5C). Comparison of the OTU richness between the original and the treated mats showed a remarkable increase after incubation with and without Cr(VI) (Figure 5D). The average number of OTUs was slightly higher in the mats without than in those with Cr(VI) (Figure 5D).

The most noticeable community shift in mats without Cr(VI) was the increase in the relative abundance of Clostridia and Bacilli from <1% to 44% and 66% in Nakhl-1 and Nakhl-2, respectively (Figure 6). Majority of these sequences fell into *Clostridium, Acetivibrio* and *Bacillus* in both sites (Figure 7). In Nakhl-1 mats without Cr(VI), the relative abundance of Betaproteobacteria exhibited a slight increase (up to 5–10%) while that of Alpha- and Gammaproteobacteria decreased (Figure 6). In Nakhl-2 mats without Cr(VI), Alphaproteobacteria showed an increase in relative abundance especially in Nakhl-2B mat (68%) and most of the alphaproteobacterial sequences belonged to the genera *Azospirillum* and *Brevundimonas* (Figure 7). After incubation with 1 mg l^–1^ Cr(VI), the growth of Alphaproteobacteria and Verrucomicrobiae was most favored in Nakhl-1 mats (53–68%) with most sequences belonging to the genera *Rhizobium*, *Brevundimonas* and *Verrucomicrobium* (Figures 6, 7). However, in Nakhl-2 mats, bacterial communities shifted in the favor of Verrucomicrobiae (16–37%) while Alphaproteobacteria and Clostridia also persisted (Figure 6). Verrucomicrobiae-related sequences mainly belonged to the genera *Verrucomicrobium* and *Luteolibacter* in Nakhl-2 mats (Figure 7).

## Discussion

### Chromium Sources in Nakhl Quarry Sump

The minerals detected in Nakhl-1 quarry, including serpentine, amphiboles, pyroxeme and chromite, have been previously described as sources of Cr(III) (Morrison et al., 2009; Mills et al., 2011; Moraetis et al., 2012; Kelepertzis and Stathopoulou, 2013). Moreover, the detection of high levels of nitrate suggests the occurrence of nitrification in this quarry, which may produce H^+^ and also contribute to the release of Cr(III) (Mills et al., 2011). However, since most of the detected chromium in Nakhl-1 quarry sump was in the form of Cr(VI), this could be attributed to the inorganic conversion of Cr(III) to Cr(VI) due to the presence of Mn-oxide minerals (Robles-Camacho and Armienta, 2000; Fandeur et al., 2009). These minerals are normally released by weathering of pyroxenes, which were detected in Nakhl-1 quarry (Kazakis et al., 2015). Cr(VI) concentration in Nakhl-1 (40 ± 1 μg l^–1^) was as high as in alluvial fans (30–75 μg l^–1^) (Ball and Izbicki, 2004; Morrison et al., 2009; Moraetis et al., 2012). Interestingly, most alluvial fans are characterized by high levels of nitrate, which were also detected in Nakhl-1 sump. These high nitrate levels could stimulate the growth and development of microbial mats.

In order to construct the balance of chromium in the Nakhl-1 quarry sump, we assumed that the release and uptake of chromium are in equilibrium. In addition, we ascribed that chromium is released through the quarry sump walls and uptake is operated through adsorption/precipitation onto the mud sediments and in the microbial mats (Figure 1E). The part of chromium remained in dissolution was equal to approximately 322 g (taking into consideration the quarry sump volume). So, if the dry density of the microbial mat would be 400–600 g/m^2^ (Sundbäck et al., 1996) and the coverage area of these mats in the quarry sump would be 50 × 70 m, then the total microbial mat mass would be 21 kg. Since the measured total amount of chromium in the microbial mats was 1,911 ± 100 mg kg^–1^ (Supplementary Table S1), 27 to 40 g of chromium may have been stored in these mats, amounting to 9 to 12% of the total chromium in the Nakhl-1 quarry sump. Most of the incorporated chromium (i.e., 95%) was firmly bound to the mats hence, indicating their important role in chromium adsorption.

### Removal of Chromium by Microbial Mats

Our study demonstrated a significant role of microbial mats from the quarry sumps in the removal of Cr(VI). A clear decline in Cr(VI) levels in the site water based on spectrophotometry and the detection of Cr(III) residues based on our ICP-OES analysis indicates that most of the Cr(VI) added into the solution was not converted to Cr(III) rather incorporated into the microbial mat. SEM and EDX analyses confirmed this by the detection of low levels of chromium within the EPS of the mat. Furthermore, the inability of the autoclaved mats to remove Cr(VI) can be attributed to the fact that the EPS was disintegrated due to autoclaving (Morin, 1998; Comte et al., 2006; Jachlewski et al., 2015) and prevented Cr adsorption into the mat, despite the low concentration used (1 mg l^–1^) in our study. This suggests that the process of Cr removal is not physical rather facilitated by microbes actively secreting EPS. Previous studies have shown that when microorganisms like cyanobacteria and bacteria (*Ochrobactrum* sp.) are exposed to heavy metals, they secrete large amounts of EPS mainly constituting macromolecules with charged functional groups exhibiting adsorptive properties. This results in the EPS serving as a binding site for heavy metal ions like Cr(III) or Cr(VI) leading to complex formation (Guibaud et al., 2005; Pal and Paul, 2008; Esteve et al., 2013; Pracejus et al., 2017; Villagrasa et al., 2019). However, it is quite challenging to exactly discern the pathway of Cr(VI) breakdown within the microbial EPS owing to the low Cr(VI) concentration used. On the other hand, the detection of chromite fragments on the mat surface based on SEM analyses is expected given the fact that these are naturally available in the quarry sump and tend to adsorb firmly on the mat surface. Chromite is a placer mineral deposit that is quite dense and stable to the extent that it can chemically/physically resist intense weathering in sub-tropical or tropical regions and maintain it’s primary elemental compositions even for thousands of years (Anand and Paine, 2002). Therefore, chromite will not account for any interference with the removal of Cr(VI) investigated in our study. The absence of other minerals like clays or mineral oxides on the mat surface also excludes the occurrence of inorganic mineral-mediated reduction of Cr(VI) to Cr(III).

Our incubations under aerobic conditions and in the dark suggest that Cr(VI) removal was mostly performed by aerobic heterotrophic bacteria. Light incubations suggest that phototrophic organisms in these mats were unable to remove Cr(VI) and also lowered the removal rate of Cr(VI) by the associated heterotrophic bacteria. This is in contradiction with previous reports, which showed that cyanobacterial (e.g., *Cyanothece* spp., *Nostoc* PCC7936 and *Nostoc linckia*) and algal (e.g., *Spirogyra*) strains were capable of directly removing Cr(VI) from wastewaters (Gupta et al., 2001; Dönmez and Aksu, 2002; Han et al., 2007; Colica et al., 2010; Mona et al., 2011). Phototrophs have even been shown to play a significant indirect role in supporting the activity of pollutant-degrading heterotrophs by providing them with O_2_, fixed N and organics (Abed and Köster, 2005). Indeed, photosynthetic activities in a cyanobacterial mat was shown to stimulate Cr(VI) removal by continuously supplying electrons (Shukla et al., 2012). The observed decrease in Cr(VI) removal by Nakhl-1 and Nakhl-2 mats in the light could be due to the growth of heterotrophic bacteria on photosynthetic exudates. Furthermore, photosynthetic activities by oxygenic phototrophs are known to increase pH in microbial mats (Abed et al., 2007), which could have influenced Cr(VI) removal. In fact, numerous studies on Cr(VI)-reducing strains consistently demonstrated a decrease in Cr(VI) removal and adsorption as pH increases (Liu et al., 2006; Silva et al., 2009; Raaman et al., 2012; Zheng et al., 2015). Optimum pH for Cr(VI) removal was variable, depending on the strain, but was generally below 9. The pH influences Cr(VI) removal by affecting adsorption/desorption of chromium ions and protonation/deprotonation of functional groups on the bacterial cell wall (Silva et al., 2009). The increase in pH results in an increase in the negative charge on the cell surface due to deprotonation of the metal binding sites, hence declining rates of metal removal (Silva et al., 2009).

### Bacterial Diversity in the Original Mats

MiSeq analysis provided a comprehensive overview of the bacterial community composition in the studied Cr(VI) contaminated microbial mats. To the best of our knowledge, next generation sequencing has been used to reveal the diversity of bacteria that can resist or tolerate heavy metals such as iron and copper (Wong et al., 2015, 2018), but rarely used to study bacterial diversity in Cr-polluted ecosystems (Desai et al., 2009; Ruvindy et al., 2016; Yu et al., 2016; Jordaan et al., 2019). Most previous studies were either cultivation-based or using other molecular techniques (e.g., cloning, fingerprinting) (Desai et al., 2009; Yu et al., 2016). Nevertheless, and regardless of the used techniques, previous studies on Cr(VI) contaminated soils also showed the existence of the same bacterial groups encountered in our mats such as Proteobacteria, Firmicutes, Actinobacteria, and Verrucomicrobiae. Since chromium has deleterious effects on both heterotrophic and phototrophic bacteria (Cervantes et al., 2001; Viti and Giovannetti, 2001; Ackerley et al., 2006), the detection of diverse bacteria in our mats suggests their possible tolerance to chromium. Proteobacteria and Firmicutes have been previously detected in Cr(VI) impacted ecosystems (Ackerley et al., 2006; Desai et al., 2009; He et al., 2014; Jordaan et al., 2019). While Firmicutes and Proteobacteria constituted 2–36% to 16–67%, respectively, of the total number of sequences in Nakhl and Samail mats, they made up to 31–53% and 18–47% in samples from chromium-polluted industrial landfill sites, respectively (Desai et al., 2009). Indeed, most of the previously isolated bacteria with the capability to remove Cr(VI) belonged to Proteobacteria and Firmicutes (Desai et al., 2009; Jin et al., 2012; Yung et al., 2014; Voica et al., 2016). For instance, *Rhizobium leguminosarum* could remove 77.3 ± 4.3% of 26 μg l^–1^ Cr (VI) in 24 h (Yung et al., 2014). The strain *Gluconacetobacter hansenii* was capable of NADH-dependent removal of Cr(VI) under aerobic conditions with the involvement of a putative chromate reductase enzyme (Shakoori et al., 2000). Many *Bacillus* strains were capable of reducing Cr(VI) at concentrations as high as 1500 mg l^–1^ (Camargo et al., 2003; Liu et al., 2008; Verma et al., 2009; Zheng et al., 2015; Wang et al., 2016; Upadhyay et al., 2017). In fact, the first purified and partly characterized chromate reductase was from a *Bacillus* strain (Park et al., 2000). Although species belonging to these genera have been detected in our mats, we have no direct proof that they are capable of removing Cr(VI) until pure isolates are obtained.

Actinobacteria, which was mostly detected in our mats from Nakhl-2 and Samail quarry sumps, have also been previously found in chromium-polluted soils (Camargo et al., 2005; Desai et al., 2009). This bacterial group is metabolically diverse, represents an important component of the microbial communities in soils and plays a vital role in the decomposition of organic matter and recycling of nutrients (Colin et al., 2013). Several Gram-positive actinobacteria such as the genus *Streptomyces* have been shown to possess the ability to bioaccumulate and/or reduce Cr(VI) (Das and Chandra, 1990; Amoroso et al., 2001; Pattanapipitpaisal et al., 2001b; Laxman and More, 2002; Desjardin et al., 2003; Pal et al., 2005; Rene’ et al., 2006; Polti et al., 2009; Prince et al., 2010). The strain *Streptomyces* sp. MC1 showed an ability to produce a bioemulsifier and to reduce 90% of Cr (VI) in soil samples amended with 50 mg kg^–1^ after 1 week of incubation without any pretreatment (Branco et al., 2005; Polti et al., 2011). This strain was successfully used together with *Zea Mays* for the bioremediation of soils contaminated with Cr(VI) (Branco et al., 2005). Isolation of Cr(VI)-reducing strains belonging to other actinobacterial genera such as *Aciditerrimonas* and *Rubrobacter*, that were mostly detected in our Nakhl-2 and Samail mats, have not been reported in previous studies.

### Shifts in Bacterial Communities

Our results corroborate previous reports detecting compositional shifts in bacterial communities after contamination with chromium (Pernthaler and Amann, 2005; Sheik et al., 2012). It is well known that there is a “bottle effect” which causes changes in microbiological processes and bacterial community structure during confined incubation of microbial samples in bottles (Ellis et al., 2003; Hammes et al., 2010; Abed et al., 2015). This bottle effect is attributed to the fact that some bacterial populations are subdominant in the field, but replace dominant populations when incubated in the laboratory. Although previous studies have documented a decrease in bacterial diversity after heavy-metal contamination (Sandaa et al., 1999; Altaf et al., 2008), This was not the case after incubating Nakhl-1 and Nakhl-2 mats with Cr(VI). Although Alphaproteobacteria were found in the original mats, sequences belonging to *Rhizobium* and *Brevundimonas* exhibited higher relative abundance in the presence of Cr(VI). Indeed, several *Rhizobium* and *Brevundimonas* spp. with the ability to resist heavy toxic metals including chromium have been isolated (Casella et al., 1988; Mamaril et al., 1997; Yung et al., 2014; Wang et al., 2016). *Brevundimonas* spp. were shown to be the main group of Cr(VI) tolerant bacteria in the magnetite mine drainage water from Hebei China and could reduce up to 350 mg l^–1^ of Cr(VI) (Li et al., 2011). While the class Verrucomicrobiae constituted 18 to 43% and 16 to 37% of total sequences in Nakhl-1 and Nakhl-2 mats incubated in the presence of Cr(VI), respectively, this class made up less than 1% of total sequences in these mats when incubated without Cr(VI). Although sequences belonging to Verrucomicrobiae have been encountered in several heavy metal contaminated ecosystems (Abulencia et al., 2006; Vishnivetskaya et al., 2011; Berg et al., 2012; Hong et al., 2015), there are so far no Cr(VI)-reducing representative strains from this group. Thus, future research should focus on obtaining novel strains of Verrucomicrobiae with the ability to remove Cr(VI).

## Conclusion

We conclude that the microbial mats in Nakhl and Samail quarry sumps were able to decrease Cr(VI) concentration under aerobic conditions and, thus, playing a vital role in chromium removal. These mats harbored diverse microbial communities, with the dominance of the bacterial classes Proteobacteria, Firmicutes, Actinobacteria and Verrucomicrobiae and these communities shifted in the favor of Alphaproteobacteria and Verrucomicrobiae when incubated in the presence of Cr(VI). Further research should attempt isolating aerobic Cr(VI)-reducing bacteria from these mats.

## Data Availability Statement

The 16S datasets generated for this study can be found in the European Nucleotide Archive (ENA), https://www.ebi.ac.uk/ena/data/view/PRJEB33895.

## Author Contributions

RA designed the project, analyzed the MiSeq data, and wrote the manuscript. MS, RA, and DM collected the samples. MS performed the chromium removal experiments and together with TM did the molecular work. ZA-R, BP, and DM performed the geochemical analysis. All authors read and corrected the manuscript.

## Conflict of Interest

The authors declare that the research was conducted in the absence of any commercial or financial relationships that could be construed as a potential conflict of interest.

## Footnotes

1 www.mrdnalab.com

2 www.ncbi.nlm.nih.gov

3 http://rdp.cme.msu.edu
