# Supplementary material for: The Role of Microbial Mats in the Removal of Hexavalent Chromium and Associated Shifts in Their Bacterial Community Composition
Source: Front Microbiol. 2020 Jan 29;11:12. doi: 10.3389/fmicb.2020.00012 (PMC7001535; doi:10.3389/fmicb.2020.00012)
Supplement: Supplementary file 1 [file Table_1.docx]

**Table S1**

| **Table S1**: Bulk chemical and qualitative mineralogical analyses of Nakhl quarry sump | | | | | | |
| --- | --- | --- | --- | --- | --- | --- |
| Sample type | Harzburgite | Weather harzburgite | Alteration veins with secondary minerals | Harzburgite rich in chromite | Mud from the bottom of the lake | Algal mat |
|  |  |  |  |  |  |  |
| MgO (%) | 13.0 | 10.9 | 7.8 | 13.9 | 8.4 | 1.3 |
| Al_2_O_3_ (%) | 0.4 | 0.4 | ND | 3.7 | 1.1 | 0.6 |
| SiO_2_ (%) | 33.2 | 45.0 | 27.9 | 44.4 | 37.1 | 8.3 |
| SO_2_ (%) | 0.4 | 0.1 | 0.1 | 0.2 | 1.2 | 3.4 |
| K_2_O (%) | ND | ND | ND | ND | 0.1 | 0.4 |
| CaO (%) | 1.3 | 2.5 | 16.9 | 2.0 | 6.7 | 4.4 |
| TiO_2_ (%) | ND | 0.01 | ND | 0.3 | 0.1 | 0.1 |
| Fe_2_O_3_ (%) | 23.0 | 14.1 | 5.2 | 18.7 | 11.0 | 3.0 |
| P (mg/kg) | ND | ND | ND | ND | ND | 350 |
| Mn (mg/kg) | 886 | 705 | 350 | 1,477 | 713 | 139 |
| Ni (mg/kg) | 1830 | 1660 | 746 | 1802 | 1218 | 403 |
| Cr (mg/kg) | 2120 | 2445 | 583 | 41124 | 12574 | 1911 |
| LOI% | 11.0 | 15.1 | 28.8 | 10 | 21 | 86 |
| Dolomite |  | ✓ |  | ✓ | ✓ | NA |
| Clinochlore |  |  | ✓ |  | ✓ | NA |
| Lizardite | ✓ | ✓ | ✓ | ✓ | ✓ | NA |
| Chrysotile | ✓ |  | ✓ | ✓ | ✓ | NA |
| Olivine | ✓ | ✓ | ✓ |  | ✓ | NA |
| Enstatite-Pyroxene | ✓ |  |  |  |  | NA |
| Forsterite-Pyroxene | ✓ |  |  |  |  | NA |
| Magnesite |  |  |  | ✓ |  | NA |
| Magnetite |  |  | ✓ |  | ✓ | NA |

ND: not detected; NA: not analyzed
