# Supplementary material for: The Role of Microbial Mats in the Removal of Hexavalent Chromium and Associated Shifts in Their Bacterial Community Composition
Source: Front Microbiol. 2020 Jan 29;11:12. doi: 10.3389/fmicb.2020.00012 (PMC7001535; doi:10.3389/fmicb.2020.00012)
Supplement: Supplementary file 2 [file Table_2.docx]

**Table S2**

**Table S2**: Estimation of total Cr content (data presented as means ± standard deviation) using ICP-OES analysis of the water samples collected after one week of aerobic incubation of Nakhl-1 and Nakhl-2 mats (A, B and C in each site). The %Cr remaining in the water as Cr(III) is calculated by subtracting the Cr(VI) content measured using spectrophotometry from the total Cr content. In case of Nakhl-2 mats (A, B and C), Cr(III) could not be detected since the remaining Cr is in the form of Cr(VI) as indicated by spectrophotometry in Figure 2

|  |  |  |  |
| --- | --- | --- | --- |
| Sample | Total Cr (mg l^-1^) | %Cr remaining in water as Cr(III) | %Cr(VI) incorporated in the mat |
| Nakhl-1A | 0.022 ± 0.009 | 3 | 97.0 |
| Nakhl-1B | 0.035 ± 0.007 | 4.3 | 95.7 |
| Nakhl-1C | 0.022 ± 0.008 | 1.7 | 98.3 |
| Nakhl-2A | 0.087 ± 0.009 | ND | 83.3 |
| Nakhl-2B | 0.062 ± 0.01 | ND | 86.6 |
| Nakhl-2C | 0.127 ± 0.031 | ND | 87.5 |
| ND: not detected | |  |  |
