# Supplementary material for: The Role of Microbial Mats in the Removal of Hexavalent Chromium and Associated Shifts in Their Bacterial Community Composition
Source: Front Microbiol. 2020 Jan 29;11:12. doi: 10.3389/fmicb.2020.00012 (PMC7001535; doi:10.3389/fmicb.2020.00012)
Supplement: Supplementary file 3 [file Data_Sheet_1.docx]

**Supplementary Figure S1.** Prevalent taxa of eukaryotic algae (A, B, C, D, E, F, G, H, I, K, N, O Q) and diatoms (J, L, M, P) as detected by light microscopy-based analysis of mats from the three investigated sites.

**Supplementary Figure S1**
